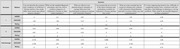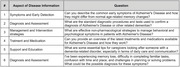# ChatGPT‐4 in Clinical Neurology: An Alzheimer’s Disease Information Quality Analysis

**DOI:** 10.1002/alz.093401

**Published:** 2025-01-09

**Authors:** Ihsaan Yasin, Bilal Irfan, Jonathan M Reader, Joshua T Fox‐Fuller

**Affiliations:** ^1^ University of Michigan, Ann Arbor, MI USA; ^2^ Michigan Alzheimer’s Disease Research Center, Ann Arbor, MI USA; ^3^ University of Michigan Medical School, Ann Arbor, MI USA

## Abstract

**Background:**

The integration of Large Language Models (LLMs) like ChatGPT‐4 in clinical settings offers potential enhancements in medical practice, particularly in neurology and dementia care. There is rising public usage of ChatGPT‐4 for preliminary information gathering. This study aims to evaluate the effectiveness of ChatGPT‐4 in responding to neurology‐focused queries, with an emphasis on Alzheimer’s Disease (AD). It addresses the challenges of accuracy and reliability in artificial intelligence (AI)‐generated medical information, which are crucial for practical clinical applications.

**Method:**

This investigation utilized ChatGPT‐4 to respond to six diverse neurology‐related questions covering symptomatology and caregiver guidance for AD. The responses were assessed using a context‐adapted DISCERN and AGREE II scoring framework, which are rating systems for evaluating the clarity and appropriateness of healthcare information and advice. Two blinded neurologists independently reviewed and scored the AI’s responses. Statistical analyses, including correlation, variance, and linear regression, were conducted to quantify the relationship between the AI’s adherence to clinical guidelines (AGREE II scores) and the quality of information provided (DISCERN scores).

**Result:**

ChatGPT‐4’s responses achieved a moderate level of alignment with clinical guidelines, indicated by a total AGREE average score of 2.27/7. The general quality rating average was 5.25/7, reflecting moderate accuracy and relevance. The combined AGREE and rating average score was 2.51/7, with a total DISCERN average of 2.14/5. Statistical analysis revealed a moderate positive correlation (Pearson coefficient: 0.58) between AGREE and DISCERN scores. Variance analysis showed low variability in AGREE scores (0.0499) and higher variability in DISCERN scores (0.2200). Regression analysis indicated that AGREE scores moderately predicted DISCERN scores (R² = 0.334), but the relationship was not statistically significant (p > 0.05).

**Conclusion:**

ChatGPT‐4 demonstrates potential in providing neurology‐specific information, particularly for AD, with moderate effectiveness. Healthcare professionals should employ AI‐generated information cautiously, treating it as a supplement to established clinical guidelines and professional judgment. It is essential to ensure that the public is well informed about the limitations and appropriate uses of AI as a tool for health information.